# Supplementary material for: Discovery and pharmacophoric characterization of chemokine network inhibitors using phage-display, saturation mutagenesis and computational modelling
Source: Nat Commun. 2023 Sep 16;14:5763. doi: 10.1038/s41467-023-41488-z (PMC10505172; doi:10.1038/s41467-023-41488-z)
Supplement: Supplementary file 4 — Source Data [file 41467_2023_41488_MOESM4_ESM.zip › SourceData/scripts/xattune_20230807170606.tar!/xattune/analysis/attune_ci/SV_R4_CCL7_HD2_plot.pdf]

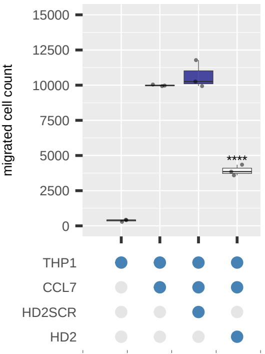
migrated cell count
15000
12500
10000
7500
5000
2500
0
****
THP1
CCL7
HD2SCR
HD2
